# Revolutionizing Sleep Health: The Emergence and Impact of Personalized Sleep Medicine

**DOI:** 10.3390/jpm14060598

**Published:** 2024-06-04

**Authors:** Sergio Garbarino, Nicola Luigi Bragazzi

**Affiliations:** 1 Department of Neuroscience, Rehabilitation, Ophthalmology, Genetics and Maternal/Child Sciences (DINOGMI), University of Genoa, 16126 Genoa, Italy; sgarbarino.neuro@gmail.com; 2 Post-Graduate School of Occupational Health, Università Cattolica del Sacro Cuore, 00168 Rome, Italy; 3 Laboratory for Industrial and Applied Mathematics (LIAM), Department of Mathematics and Statistics, York University, Toronto, ON M3J 1P3, Canada; 4 Human Nutrition Unit (HNU), Department of Food and Drugs, University of Parma, 43125 Parma, Italy

**Keywords:** sleep, sleep health, personalized medicine, artificial intelligence, machine learning, deep learning

## Abstract

Personalized sleep medicine represents a transformative shift in healthcare, emphasizing individualized approaches to optimizing sleep health, considering the bidirectional relationship between sleep and health. This field moves beyond conventional methods, tailoring care to the unique physiological and psychological needs of individuals to improve sleep quality and manage disorders. Key to this approach is the consideration of diverse factors like genetic predispositions, lifestyle habits, environmental factors, and underlying health conditions. This enables more accurate diagnoses, targeted treatments, and proactive management. Technological advancements play a pivotal role in this field: wearable devices, mobile health applications, and advanced diagnostic tools collect detailed sleep data for continuous monitoring and analysis. The integration of machine learning and artificial intelligence enhances data interpretation, offering personalized treatment plans based on individual sleep profiles. Moreover, research on circadian rhythms and sleep physiology is advancing our understanding of sleep’s impact on overall health. The next generation of wearable technology will integrate more seamlessly with IoT and smart home systems, facilitating holistic sleep environment management. Telemedicine and virtual healthcare platforms will increase accessibility to specialized care, especially in remote areas. Advancements will also focus on integrating various data sources for comprehensive assessments and treatments. Genomic and molecular research could lead to breakthroughs in understanding individual sleep disorders, informing highly personalized treatment plans. Sophisticated methods for sleep stage estimation, including machine learning techniques, are improving diagnostic precision. Computational models, particularly for conditions like obstructive sleep apnea, are enabling patient-specific treatment strategies. The future of personalized sleep medicine will likely involve cross-disciplinary collaborations, integrating cognitive behavioral therapy and mental health interventions. Public awareness and education about personalized sleep approaches, alongside updated regulatory frameworks for data security and privacy, are essential. Longitudinal studies will provide insights into evolving sleep patterns, further refining treatment approaches. In conclusion, personalized sleep medicine is revolutionizing sleep disorder treatment, leveraging individual characteristics and advanced technologies for improved diagnosis, treatment, and management. This shift towards individualized care marks a significant advancement in healthcare, enhancing life quality for those with sleep disorders.

## 1. Introduction

Sleep is a ubiquitous natural phenomenon [1,2], yet extremely complex [3], unique [2,3], and with a significant amount of inter- and intra-individual variability [4,5,6,7,8], which has been found to correlate with age, race/ethnicity, body mass index (BMI), physical and mental health, and chronotype [9], among others.

By embracing such a complexity, “personalized sleep medicine” represents a cutting-edge, deeply transformative field in healthcare that caters to the specific sleep-related healthcare needs of individuals, considering the bidirectional relationship between sleep and health [10]. This novel framework goes beyond traditional one-size-fits-all approaches and underscores the distinct physiological and psychological characteristics of each person to optimize sleep quantity and quality and effectively address sleep disorders [11].

The foundation of this approach lies in recognizing and responding to a range of diverse factors that influence sleep, such as genetic predispositions, behavior, lifestyle habits (nutrition, physical activity, and use of drugs and stimulants), environmental influences, and underlying health conditions [12,13]. By considering these variables, personalized sleep medicine can offer more accurate diagnoses, targeted treatments, and proactive management strategies that better fit each individual’s needs.

The rapid development and integration of new technologies in this field are noteworthy. The advent of new tools, including digital ones, and a deeper understanding of sleep patterns are propelling personalized sleep medicine to the forefront, offering innovative solutions for sleep-related issues [14]. Wearable devices, mobile health applications, and advanced diagnostic tools are increasingly employed to gather, store, and process detailed data on sleep patterns, quantity, quality, and disturbances. These technologies enable continuous monitoring and analysis, providing a wealth of information that can be used to tailor treatments and interventions effectively [15,16,17].

Moreover, the advent of sophisticated data analysis techniques, particularly machine learning (ML) and artificial intelligence (AI), including generative conversational AI [18], has further enhanced and augmented the capabilities of personalized sleep medicine [19,20,21]. These technologies facilitate the interpretation of large datasets, revealing nuanced insights into sleep behaviors and potential disorders. They can empower healthcare providers to craft highly customized treatment plans that align closely with an individual’s specific sleep profile [22].

Additionally, a deeper understanding of sleep science, including the study of circadian rhythms and sleep physiology, plays a crucial role in this field [23]. Research in these areas is uncovering new layers of complexity in how sleep affects overall health and well-being, contributing valuable knowledge that can inform personalized treatment approaches [24]. In essence, personalized sleep medicine stands in the vanguard of a healthcare revolution, one that emphasizes individualized care and harnesses the power of technology and scientific knowledge to improve sleep health [25].

This approach not only addresses sleep disorders more effectively but also enhances the overall quality of life, recognizing that good sleep is a cornerstone of health and well-being [26].

The research aim of the present review is to better frame and conceptualize the emerging field of personalized sleep medicine, envisaging how this disruptive approach in healthcare, tailoring sleep-related treatments and interventions to individual needs and leveraging new technologies, sophisticated data analyses, and a more profound and nuanced understanding of sleep science, can improve sleep health and overall well-being (Table 1).

## 2. Wearable Technology, IoT/IoMT Integration, Telemedicine, and Virtual Healthcare Platforms

The next generation of wearable technology is already offering and will offer increasingly sophisticated and non-intrusive monitoring of sleep patterns. Integration with the Internet of Things (IoT), the Internet of Medical Things (IoMT), and smart home systems could enable a more holistic approach to managing sleep environments, optimizing factors like lighting, temperature, and noise based on individual preferences and needs [27].

Telemedicine in sleep medicine and more broadly in healthcare has grown exponentially since 2015, driven initially by the need for specialty care access and advancing technologies. The SARS-CoV-2 pandemic accelerated its adoption as health systems sought safe care delivery methods amid restrictions on non-emergent care [28].

Regarding its effectiveness and cost-effectiveness, a number of reviews have underscored the nuanced benefits of telemedicine in sleep disease management and opened up discussions for its optimized use in clinical settings, alongside its impact on medical staff workload, suggesting potential benefits in healthcare resource allocation but also raising questions about its implementation.

For instance, according to a recently published systematic review and meta-analysis [29], synthesizing 33 articles and totaling 8689 participants, the impact of telemedicine-based follow-up management on patients with obstructive sleep apnea (OSA) was found to be beneficial. More in detail, telemedicine increased the average daily usage of continuous positive airway pressure (CPAP) by 36 min, raising the weighted mean difference to 0.61 (95% confidence interval: 0.39 to 0.83). It also increased the percentage of days when CPAP was used for more than four hours by 10.67%, even if telemedicine did not significantly enhance overall CPAP compliance, with an odds ratio of 1.13 (95% confidence interval: 0.72 to 1.76). The analysis showed marginal effects on sleep quality, with a standardized mean difference of 0.15 (95% confidence interval: −0.03 to 0.32) and a decrease in daytime sleepiness by a weighted mean difference of −0.26 (95% confidence interval: −0.79 to 0.28). The reduction in the apnea–hypopnea index (AHI), one of the key metrics of OSA, was −0.53 (95% confidence interval: −3.58 to 2.51). In terms of the overall quality of life, the standardized mean difference was −0.25 (95% confidence interval: −0.25 to 0.76). The conclusion drawn was that while telemedicine-based follow-up within six months helped improve CPAP usage in patients, it did not enhance sleep quality, daytime sleepiness, apnea severity, or quality of life compared to traditional follow-up methods. Additionally, it was found to be more cost-effective, although there was no consensus on whether it increased the workload for medical staff.

The areas of concern identified in this systematic review and meta-analysis as well as in other reviews should be addressed to optimize the use of telemedicine in the management of sleep diseases, such as OSA. While telemedicine has shown some advantages, it does not significantly improve or improves only marginally other sleep-related characteristics and domains. Efforts should focus on enhancing the comprehensive benefits of telemedicine, ensuring that it not only promotes device usage but also contributes to overall patient health outcomes. Further research and improvements in telemedicine protocols could explore more integrated approaches that combine technology with traditional care methods to address these gaps. Additionally, the economic and operational impacts of implementing telemedicine, such as its cost-effectiveness and potential to increase the workload for medical staff, need a thorough evaluation. By addressing these concerns, healthcare providers can better leverage telemedicine as a supportive tool in the management of sleep disorders, making it a more effective and sustainable option in clinical practice.

Finally, expanding telemedicine services and virtual healthcare platforms will make personalized sleep medicine more accessible. This will be especially beneficial for patients in remote or underserved areas, allowing them to receive specialized care without the need for physical travel [30].

For example, the TeleSleep Enterprise-Wide Initiative (EWI) represents an important, successful initiative aimed at enhancing access to sleep care for rural veterans through the development and implementation of national telehealth networks [31,32].

## 3. Advanced Data Integration and Analysis and Biomarker (Genomic and Molecular) Research

Future developments in personalized sleep medicine will likely emphasize the integration of diverse data sources, reflecting the impact of the complex, non-linear interplay of genetic, environmental, and lifestyle factors on sleep health. Advanced data analytics and AI will play a crucial role in synthesizing this information to provide more comprehensive and personalized sleep assessments and treatments [33].

The exploration of genetic and molecular aspects of sleep [34,35,36] will deepen, potentially leading to breakthroughs in understanding individual sleep disorders as well as the determinants of sleep health.

Recent advancements in the field of genomics on sleep, including genome-wide association studies (GWASs), have provided substantial insights into the genetic underpinnings of various sleep-related traits and disorders. Studies have identified numerous genetic *loci* associated with the architecture of sleep in terms of sleep duration, quality, and timing. For example, a GWAS conducted by the UK Biobank and other cohorts identified genes related to neurotransmitter regulation, such as *PAX8*, which influence sleep duration, suggesting that genetic factors can significantly affect how long and how well individuals sleep [37].

Other GWASs on insomnia have identified several risk *loci*, highlighting the involvement of brain regions and pathways that regulate sleep, stress responses, and circadian rhythm. For instance, variants in genes like *MEIS1* and others that are also implicated in neurodevelopmental and psychiatric disorders suggest a complex interplay between sleep disruption and mental health [38].

Studies focusing on circadian rhythms have discovered genes that affect both the timing of sleep and physiological processes that follow a daily cycle. Notably, variants near genes such as *PER1* and *CRY2* are significant, underscoring the genetic basis of circadian regulation that can influence sleep patterns and overall health [39,40].

Specific sleep disorders like narcolepsy have been linked to genetic variants through GWASs. A notable example is the identification of a strong association between narcolepsy and variants near the *HLA-DQB1* and *HLA-DQA1* genes [41], highlighting immune system involvement and underscoring the bidirectional relationship between sleep and immunity [42,43].

Recently, some multi-trait GWASs have started to explore the genetic correlations between sleep traits and other health outcomes, besides psychiatric disorders, such as metabolic health and cardiovascular disease. These studies suggest that poor sleep can be both a cause and consequence of other health issues, driven by shared genetic pathways [44,45].

Furthermore, comparative studies across different populations, including cross-population and multi-ethnic/multi-ancestry meta-analyses, have begun to reveal how genetic determinants of sleep traits may vary by ancestry, which is critical for understanding the global epidemiology of sleep disorders and tailoring interventions appropriately. These insights from GWASs are crucial for developing personalized medicine approaches and could lead to better therapeutic strategies targeting sleep disorders and related health conditions [46,47].

Finally, a recently published study [48] utilized Genomic Structural Equation Modeling (Genomic SEM) to explore the genetic foundations of sleep health and its links to psychopathology. This method involves using diverse GWAS data to construct models estimating the genetic relationships and latent variables among various sleep-related traits. By comparing different genetic models, the study identified how multiple dimensions of sleep health genetically correlate and interact with psychological well-being and psychiatric disorders, providing insights into potential targets for improving mental health outcomes. More specifically, six sleep health factors (namely, sleep efficiency, sleep duration, sleep regularity, daytime alertness, non-insomnia, and circadian preference) showed significant genetic correlations with each other and with various psychopathological conditions, highlighting that these sleep health components are genetically distinct yet interrelated, and they differently associate with psychological and psychopathological outcomes. Taken together, these findings underscore the complexity of sleep as a multivariate health behavior with implications for mental health, suggesting that interventions targeting specific sleep health components could differentially influence multiple health outcomes [49].

All this could pave the way for highly personalized treatment plans based on genetic predispositions and molecular pathways: as research continues, the integration of GWAS data with other biological data (like epigenetics and gene expression studies) [50] is likely to deepen our understanding of sleep and its complex relationship with human health.

## 4. Personalized Sleep Stage Estimation and Circadian Rhythm Analysis

Accurate sleep stage estimation is essential for the effective diagnosis and management of sleep disorders. Recently, there have been significant advancements in the methodologies used to assess sleep stages, including the adoption of non-contact devices [51,52] and techniques based on ML and deep learning [53]. These innovations enhance the precision of sleep stage detection, which is crucial for determining the overall quality and quantity of sleep, as well as its various impacts on health. The integration of ML and deep learning in sleep medicine is transformative: these technologies analyze large datasets to reveal patterns and anomalies that were previously difficult or impossible to detect. This capability allows for a deeper understanding of sleep processes and aids in the identification of specific sleep disorders. By providing a clearer picture of an individual’s sleep patterns, these advanced analytical tools can lead to better-targeted and more effective treatments [53,54]. ML and deep learning are particularly advantageous in that they allow for personalized sleep stage estimation. These technologies can adapt to the unique sleep patterns of individuals, offering a customized analysis that aligns closely with personal sleep characteristics. This individualized approach is critical in optimizing treatment strategies for sleep disorders, potentially improving clinical outcomes [53,54].

For instance, in a recent study [54], the authors crafted a personalized sleep intervention framework leveraging real-world sleep–wake data from wearable technology to enhance alertness during designated times. This innovative framework employed a mathematical model to monitor dynamic sleep pressure (homeostatic process or process S) and circadian rhythms (process C) [55], drawing on the user’s sleep history to provide accurate real-time alertness predictions, even for shift workers with irregular sleep and work schedules. This approach enabled the identification of a novel sleep–wake pattern known as adaptive circadian split sleep (ACSS), which combines a primary sleep period with a late nap to sustain high alertness throughout both work and leisure times for shift workers. The authors also developed a mobile app integrating this model to suggest personalized sleep schedules tailored to maximizing alertness during specific activities, considering each user’s preferred sleep onset and total sleep time. This innovation not only minimizes the risk of errors during times when high alertness is needed but also enhances the health and quality of life for those with shift-work-like schedules.

Another notable application of these technologies is their use in analyzing ultradian rhythms—natural cycles within the sleep period typically lasting 60 to 120 min. One innovative study [56] explored the integration of ML algorithms with predicted ultradian rhythms to refine sleep stage estimation. This approach involves weighting the ML results according to these rhythms, enhancing the system’s ability to adapt to individual sleep cycle variations. Validated through experiments involving human subjects, this method demonstrated its efficacy by producing results that were competitive with, or superior to, traditional ML techniques. This highlights its potential clinical relevance and the value of incorporating such rhythms into sleep analysis models. The focus on individual sleep patterns and the adjustment for ultradian rhythms illustrate a significant shift towards more customized healthcare solutions in sleep medicine.

These advancements highlight the potential of personalized sleep medicine, where treatment is tailored not just to the specific sleep disorder but to the individual characteristics and sleep profile of each patient. As ML and deep learning continue to evolve, their impact on the field of sleep medicine is likely to grow, leading to more precise diagnostics, better patient outcomes, and a deeper understanding of the complexities of sleep.

## 5. Computational Models of Sleep Disorders

Computational models represent a cutting-edge approach in the customized treatment of sleep disorders: these models offer a sophisticated method of personalizing management plans based on individual patient needs and conditions [57].

By using detailed 3D models of a patient’s anatomical structures, clinicians can accurately simulate how different treatment strategies might affect the patient. For example, in OSA, such models can predict how changes in airway structure due to various interventions (like CPAP, dental appliances, or surgery) impact airflow and tissue displacement during sleep. This level of detail allows for highly personalized therapy plans that are tailored not just to the disease but to the patient’s unique physiological characteristics, as previously mentioned. Also, computational modeling can facilitate the optimization of treatment outcomes by allowing for the simulation and assessment of multiple therapeutic scenarios. This approach helps in selecting the most effective treatment strategy with potentially higher success rates and reduced side effects or complications. Further, these models can enhance the understanding of the pathophysiological mechanisms underlying sleep disorders. By observing how different variables affect sleep dynamics and respiratory patterns, researchers can identify new therapeutic targets and improve existing treatment modalities.

For instance, a study [57] explored the use of computational models for the individualized treatment of sleep disorders, such as OSA, employing patient-specific partial 3D finite element studies. This approach underscores the effectiveness of tailor-made treatment strategies, especially in managing complex sleep disorders. By adapting to the specific conditions of each patient, these computational models pave the way for more effective, customized treatment plans. Moreover, computational models can be integrated with other technologies such as ML and real-time data monitoring (from wearables or bedside monitors). Such integration can lead to dynamic models that not only predict but also adapt to changes in a patient’s condition over time, potentially leading to continuously optimized treatment plans [57].

Despite these advantages, significant challenges remain in the broader implementation of computational models. These include the need for high-quality, patient-specific data, the computational cost of detailed simulations, and the necessity for robust validation of the models against clinical outcomes. Future research should focus on addressing these challenges, improving the accuracy of the models, and integrating them more seamlessly into clinical workflows. By addressing these aspects, computational models could revolutionize the management of sleep disorders, making treatments not only more effective but also more responsive to the changing health landscape of each patient [57].

## 6. Customized Sleep Pharmacogenetics and Pharmacotherapy

Personalized sleep medicine is poised to revolutionize pharmacotherapy, with medications being tailored to individual patient profiles [58,59]. This could involve adjusting dosages and the timing of administration and choosing specific drug types based on individual responses and tolerances.

Traditional one-size-fits-all dosing strategies are often inadequate because individuals vary in their responses to medications. In personalized sleep medicine, dosages are carefully adjusted based on factors like the patient’s metabolism, age, sex/gender, BMI, and underlying health status. This ensures maximum effectiveness while minimizing potential side effects.

The timing of medication [60,61] can have a profound impact on its effectiveness, particularly in the realm of sleep disorders. Personalized sleep medicine takes into account the patient’s circadian rhythms and sleep patterns. Medications might be administered at specific times to align with these patterns, enhancing their efficacy.

Different patients may respond better to different types of sleep medications [60,61]. Some might benefit more from sedatives, while others may need medications that alter sleep architecture. Factors like underlying health conditions, concurrent medications, and even genetic makeup can influence which drug is most suitable for a patient.

Melatonin serves as a prime example of customization in sleep medications through the lens of personalized pharmacogenetics and chronotherapy. As a naturally occurring hormone that regulates the sleep–wake cycle, melatonin supplements are commonly used to treat sleep disorders such as insomnia and jet lag. However, its effectiveness can vary widely among individuals, a variation that can be attributed to genetic differences [62,63]. Research in pharmacogenetics has revealed that certain genetic variants in melatonin receptor genes, such as *MTNR1A* and *MTNR1B*, influence the efficacy of endogenous melatonin [64,65]. By analyzing these genetic markers, as well as other biomarkers, healthcare providers can tailor melatonin treatment plans to enhance effectiveness and reduce side effects, offering a more personalized approach to sleep management, representing a major shift towards individualized medicine, where treatments can be optimized based on a person’s genetic profile [66,67].

Melatonin’s role in sleep management also highlights its importance from a chronotherapy perspective. Melatonin, integral in regulating circadian rhythms, is particularly well suited for this approach. By administering melatonin at specific times, clinicians can effectively shift and re-synchronize these rhythms, thereby aiding individuals whose sleep patterns are misaligned with their environmental cues, such as shift workers or those experiencing jet lag. This approach based on strategic timing not only improves sleep quality but also harnesses the body’s clock to enhance overall well-being. Thus, melatonin exemplifies how understanding and integrating chronobiology into treatment plans can significantly improve the management of sleep disorders and potentially other circadian rhythm-related conditions [63].

Furthermore, continuous monitoring and feedback are essential components of personalized sleep medicine [68]. This could involve regular check-ups, longitudinally designed sleep studies, and the use of wearable technology to track sleep patterns and medication effects [69,70]. Adjustments to the treatment plan are made based on these observations to ensure optimal outcomes. Advances in genetics and biomarker research can provide insights into how a patient might respond to certain sleep medications. This can guide the selection of drugs and dosages for better, more personalized treatment outcomes [71].

Furthermore, personalized sleep medicine also recognizes the importance of non-pharmacological interventions. This may include tailored advice on sleep hygiene, dietary changes, and stress management techniques that can complement the pharmacological treatment [72].

Overall, personalized sleep medicine aims to move beyond the traditional trial-and-error approach, offering more precise, effective, and safer treatment options for individuals suffering from sleep disorders. This represents a significant shift towards a more patient-centered approach in healthcare.

## 7. Obstructive Sleep Apnea as a Case Study

OSA is a complex disorder characterized by repeated episodes of partial or complete obstruction of the upper airway during sleep, leading to impaired sleep, disrupted sleep patterns, and decreased oxygen levels in the blood [73,74]. OSA represents a significant global health concern, generating a dramatic epidemiological and clinical burden [75]. It is estimated, indeed, to affect nearly one billion adults worldwide with enormous individual and public health consequences. From a gender perspective, this pathology affects 17% and 34% of women and men, respectively, and excessive daytime sleepiness is its most common presenting symptom, although it is reported by as few as 15–50% of people with OSA in the general population [73,74,75,76]. The traditional one-size-fits-all approach often fails to address the multifaceted nature of OSA, which is where the concept of clinical phenotypes and endotypes (representing subtypes of a condition, defined by distinct functional or pathobiological mechanisms) becomes crucial [77,78,79,80].

Certain craniofacial structures and alignments, like retrognathia (receded jaw), high-arched palate, and overbite, can predispose individuals to OSA [81]. By taking into account these features, personalized treatment may involve dental or orthodontic interventions such as mandibular advancement devices (MADs) or surgeries to modify the craniofacial structure, thereby improving airway patency [82,83]. Skeletal features, including the size and positioning of the jaw and other facial bones, can significantly affect airway size and shape [81]. As such, personalized interventions might include orthognathic surgery to reposition skeletal components for patients with severe skeletal discrepancies [81,82,83].

The neurological control of upper airway muscles plays a role in OSA too, as reduced muscle tone during sleep can lead to airway collapse [84,85,86,87]. Personalized treatment targeting this pathophysiological mechanism could include therapies to enhance the tone and function of these muscles, possibly through neuromuscular electrical stimulation or targeted exercises [88].

Lung function and respiratory control mechanisms also contribute to OSA, particularly in individuals with underlying respiratory diseases [84,85,86,87]. Management might involve using positive airway pressure therapies, such as CPAP, to keep the airway open, or addressing underlying respiratory conditions.

Obesity, particularly central obesity, is a significant risk factor for OSA. Fat deposits around the neck and upper airway can exacerbate airway obstruction. Weight loss programs, bariatric surgery, or other metabolic interventions could be part of a personalized treatment plan for such individuals [89,90,91,92].

Personalized treatment begins with a comprehensive evaluation of the patient, including detailed physical examinations, imaging studies, and sleep studies [93]. Genetic testing and molecular profiling might also play a role in understanding individual susceptibilities and responses to treatment [94,95,96,97].

Based on the assessment, a combination of therapies may be recommended [98,99]. This could include medical devices, surgical interventions, lifestyle changes, and pharmacotherapy [100,101,102,103]. For example, a patient with a pronounced craniofacial endophenotype might benefit more from orthodontic or surgical interventions than from CPAP therapy alone. Besides clinical subtypes, comorbidities and patients’ individual preferences should be taken into account when customizing the treatment plan [104,105,106,107]. Continuous monitoring and regular follow-up are essential to assess the effectiveness of the treatment and make adjustments as needed [108]. Wearable technology and telemedicine platforms can facilitate ongoing monitoring and communication between patients and healthcare providers [108].

Existing strategic frameworks for managing OSA adopt a uniform approach that applies to all patients—the so-called “one-size-fits-all models” [105,106,107,109]. Ultimately, the diagnosis and severity of OSA are based on key indicators, like the AHI, the oxygen desaturation index (ODI), minimum oxygen saturation, the Epworth Sleepiness Scale (ESS), the duration of apneas and hypopneas, and treatment initiated with CPAP, followed by alternative treatment options (e.g., MAD) if CPAP “fails”. As previously mentioned, this will have to consider the heterogeneity of patients with OSA, characterized by different risk factors, pathophysiological causes, clinical manifestations, and comorbidities. A range of analytical methods, such as cluster analysis, can be utilized to discern patterns within clinical heterogeneity and OSA phenotypes. Identifying patient subtypes with distinct characteristics through these techniques may facilitate more tailored and effective strategies for prognosis and treatment (e.g., symptom-based subtypes such as “minimally symptomatic”, “excessive sleepiness”, and “disturbed sleep”) [106,110].

In precision medicine for OSA, polysomnographic subtypes, distinguished by the association of respiratory events with hypoxemia, arousals, or both, also present variable risks of cardiovascular disease and responses to therapy [106,110]. By linking various phenotypes to clinically relevant outcomes, a correct and rigorous assessment of phenotype reproducibility can be ensured, and the need for tools that categorize and interpret these differences effectively can be satisfied. Thus, clinicians can better tailor treatments to individual patient profiles. This approach emphasizes the importance of a nuanced understanding of each patient’s specific sleep disorder characteristics, enabling more targeted and potentially more effective therapeutic interventions.

Treating OSA effectively often requires a multidisciplinary team, including sleep specialists, dentists, surgeons, neurologists, pulmonologists, otorhinolaryngologists, and dietitians [111]. Collaboration among these specialists ensures a comprehensive approach to addressing the various endophenotypes of the patient [111].

Finally, educating patients about their specific endophenotypes and involving them in treatment decisions are crucial for adherence and success [112,113,114]. Personalized treatment plans should consider the patient’s preferences, lifestyle, and ability to adhere to the recommended therapies [115]. In summary, recognizing and addressing the multiple endophenotypes in OSA are fundamental to providing personalized and effective treatment. This approach not only targets the specific contributing factors of the disorder for each individual but also offers a more holistic and patient-centered strategy for managing this complex condition.

## 8. Future Directions

The field of personalized sleep medicine is anticipated to benefit from cross-disciplinary collaborations, involving sleep scientists, neurologists, psychologists, data scientists, bioengineers, and other experts. Such collaborations could lead to more innovative solutions and a deeper understanding of complex sleep disorders [111].

There will be an increased focus on integrating “customized” cognitive behavioral therapy (CBT) and other mental health interventions into personalized sleep medicine [113]. This holistic approach will address both the psychological and physiological aspects of sleep disorders, offering a more integrated and effective treatment strategy.

Increased public awareness and educational initiatives about the importance of personalized approaches to sleep health will be essential. Educating both healthcare providers and the general public can lead to earlier recognition of sleep disorders and more proactive management [112,113,114].

As personalized sleep medicine evolves, there will be a need for updated regulatory frameworks to address privacy, data security, and ethical considerations, especially concerning genetic and biometric data [116].

Finally, emphasis on longitudinal studies and continuous monitoring will provide deeper insights into how sleep patterns evolve over time in relation to aging, lifestyle changes, and health status, further refining personalized treatment approaches. The process of monitoring and managing sleep disorders, notably OSA, as well as ensuring that patients consistently adhere to and comply with their prescribed treatment plans, poses substantial challenges. This involves regular follow-up appointments to assess the patient’s progress, adjustments to treatment protocols based on the patient’s response, and continuous education and support to encourage patient compliance. Effective management of sleep disorders like OSA requires a multifaceted approach, including the use of medical devices such as CPAP machines, lifestyle modifications, and possibly medication. Ensuring patient engagement and adherence to these treatment strategies is crucial for the successful management of the condition and to prevent associated risks such as cardiovascular problems, daytime fatigue, and decreased quality of life. The complexity of these tasks highlights the need for a coordinated approach involving healthcare professionals, patient education, and support systems to overcome these challenges [117,118].

## 9. Conclusions

Within the broader P4–P6 (personalized, predictive, preventive, participatory, psycho-cognitive, and public) framework of contemporary medicine [119,120,121,122], “personalized sleep medicine” has great potential to revolutionize the understanding and treatment of sleep disorders. By focusing on individual characteristics and employing advanced technologies like ML and computational modeling, this field offers a more nuanced and effective approach to sleep health. The promise of personalized sleep medicine lies in its ability to provide better diagnosis, treatment, and management of sleep disorders, leading to improved sleep quality and overall health outcomes.

This shift towards individualized care is a significant step forward in the realm of healthcare, showcasing the potential of personalized approaches to enhance the quality of life for individuals with sleep disorders.

## Figures and Tables

**Table 1:** Conceptualization of personalized sleep medicine: an overview of major concepts, advancements, applications, and technologies in patient-centered sleep medicine.

Concept/Advancement/Application/Technology	Description
Personalized Approach	Tailoring sleep-related healthcare to individual needs, recognizing the unique physiological and psychological characteristics of each person
Taking into Account Different Influencing Factors	Considering genetic predispositions, lifestyle habits, environmental influences, and underlying health conditions
Technology Integration (Wearable Devices and IoT/IoMT)	Making use of sophisticated devices for non-intrusive monitoring of sleep patterns, including wearable devices, mobile health applications, and advanced diagnostic tools for detailed data collection and analysis; integration with IoT/IoMT for optimizing sleep environments
Data Analysis Techniques (Machine Learning and AI in Sleep Analysis)	Employing machine learning and AI for interpreting large datasets and providing customized treatment plans, including utilization of non-contact devices and advanced algorithms (machine learning/deep learning techniques) for personalized sleep stage estimation, analysis of sleep patterns, and circadian rhythm analysis
Circadian Rhythms and Sleep Physiology	Researching these areas and contributing to a deeper understanding of sleep’s impact on health to inform personalized treatment approaches in an evidence-based, data-driven fashion
Advanced Data Integration and Analysis	Emphasizing the integration of genetic, environmental, and lifestyle data for comprehensive sleep assessments and treatments
Biomarker (Genomic and Molecular) Research	Exploring individual sleep disorders and determinants of sleep health, leading to personalized treatment plans
Computational and Mathematical Models for Sleep Disorders, Including Patient-specific 3D Computational Models	Creating patient-specific treatments for conditions like obstructive sleep apnea using computational models
Customized Sleep Pharmacogenetics and Pharmacotherapy	Delivering personalized and data-driven insights for the selection of drugs; customizing dosages and timing of sleep medication based on genetic predispositions, molecular pathways, metabolism, and underlying health status; and providing continuous pharmacological monitoring and feedback
Customized Non-pharmacological Interventions	Incorporating advice on sleep hygiene, dietary changes, and stress management techniques to complement pharmacological treatments
Cross-disciplinary and Multidisciplinary Collaborations	Collaboration among various experts and specialists (like dentists, surgeons, neurologists, pulmonologists, otorhinolaryngologists, and dietitians) for a comprehensive approach to treating complex sleep disorders, innovative solutions, and a deeper understanding of sleep issues
Regulatory and Ethical Considerations.	Addressing concerns related to privacy, data security, and the use of genetic/biometric data
Educational Initiatives and Public Awareness	Increasing awareness about personalized sleep health, leading to earlier recognition and proactive management of sleep disorders
Compliance and Patient Engagement	Ensuring adherence to treatment plans through education, support systems, and regular follow-up
Longitudinal Studies and Continuous Monitoring	Providing insights into the evolution of sleep patterns over time, refining personalized treatment approaches
Telemedicine and Virtual Healthcare Platforms	Expanding services for accessible personalized sleep medicine, especially beneficial for remote or underserved areas.
Holistic and Integrative Approaches	Integrating mental health interventions and cognitive behavioral therapy in sleep medicine

## Data Availability

Not applicable.
